# Ros3 (Lem3p/CDC50) Gene Dosage Is Implicated in Miltefosine Susceptibility in *Leishmania (Viannia) braziliensis* Clinical Isolates and in *Leishmania (Leishmania) major*

**DOI:** 10.1021/acsinfecdis.0c00857

**Published:** 2021-03-16

**Authors:** Caroline R. Espada, Andreia Albuquerque-Wendt, Valentín Hornillos, Eva Gluenz, Adriano C. Coelho, Silvia R. B. Uliana

**Affiliations:** † Departamento de Parasitologia, Instituto de Ciências Biomédicas, Universidade de São Paulo, São Paulo, Brazil; ‡ Sir William Dunn School of Pathology, University of Oxford, Oxford, United Kingdom; § Global Health and Tropical Medicine, Instituto de Higiene e Medicina Tropical, Universidade de Lisboa, Lisboa, Portugal; ∥ Departamento de Química Orgánica, Universidad de Sevilla and Centro de Innovación en Química Avanzada, Sevilla, Spain; ⊥ Wellcome Centre for Integrative Parasitology, Institute of Infection, Immunity & Inflammation, College of Medical Veterinary and Life Sciences, University of Glasgow, Glasgow, United Kingdom; # Departamento de Biologia Animal, Instituto de Biologia, Universidade Estadual de Campinas, Campinas, Brazil

**Keywords:** *Leishmania braziliensis*, miltefosine, drug resistance, clinical isolates, CRISPR/Cas9, treatment

## Abstract

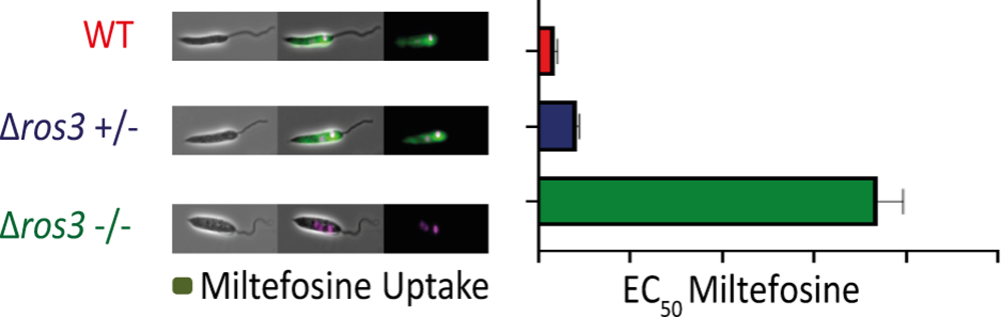

The Ros3 protein is a component of the MT-Ros3 transporter complex, considered as the main route of miltefosine entry in *Leishmania*. *L. braziliensis* clinical isolates presenting differences in miltefosine susceptibility and uptake were previously shown to differentially express *ros3*. In this work, we showed that the *ros3* gene copy number was increased in the isolate presenting the highest rates of miltefosine uptake and, thus, the highest susceptibility to this drug. The role of the *ros3* gene dosage in miltefosine susceptibility was then investigated through a modulation of the gene copy number using two distinct approaches: through an overexpression of *ros3* in a tolerant *L. braziliensis* clinical isolate and in *L. major* and by generating mono- and diallelic knockouts of this gene in *L. major* using clustered regularly interspaced short palindromic repeats (CRISPR) Cas9 (Cas = CRISPR-associated). Although the levels of *ros3* mRNA were increased at least 40-fold in overexpressing clones, no significant reduction in the half-maximal effective concentration (EC_50_) for miltefosine was observed in these parasites. The partial or complete deletion of *ros3* in *L. major*, in turn, resulted in a significant increase of 3 and 20 times, respectively, in the EC_50_ to miltefosine. We unequivocally showed that the *ros3* copy number is one of the factors involved in the differential susceptibility and uptake of miltefosine.

*Leishmania (Viannia) braziliensis* is the main etiological agent of tegumentary leishmaniasis in Brazil.^1,2^ Infections caused by this species predominately manifest as localized cutaneous lesions but can also cause a severe mucosal disease or disseminated cutaneous manifestations.^3^ Therefore, a systemic treatment in these cases is mandatory, and the limitations of the treatment options in use in Brazil become even more alarming.^4^ The current therapy for leishmaniasis in Brazil relies on pentavalent antimonial and amphotericin B, both of them highly toxic and parenterally administered.^5^ Moreover, in some regions of Brazil the cure rates for cutaneous leishmaniasis (CL) upon treatment with meglumine antimoniate have drastically dropped to ∼50%.^6,7^ For all these reasons, alternative therapies are highly needed in order to overcome these limitations.

Miltefosine (MF) is currently the most effective oral drug available for leishmaniasis treatment.^8^ In Brazil, recommendations for the use of MF for CL treatment were issued in 2018, but the drug is still not available for clinical use.^9^ Two clinical trials employing MF for CL treatment in patients infected with the *Leishmania (Viannia)* species of two different regions of Brazil showed cure rates of ∼70%.^6,10^ Although MF is not devoid of side effects, those are mostly milder than the side effects of antimony and amphotericin.^11^ Being the only oral drug in clinical use, MF is of great importance for leishmaniasis chemotherapy. However, the significant drop in cure rates observed in visceral leishmaniasis patients in India, together with the recent isolation of resistant parasites from patients previously treated with MF, raised an alarm on the possible loss of this drug due to the selection of resistance.^12−16^ Moreover, recently data characterizing the in vitro susceptibility of *L. infantum* Brazilian clinical isolates recovered from patients enrolled in a clinical trial with MF for VL treatment revealed an alarming correlation between treatment failure and the intrinsic parasite susceptibility to MF.^17^

Miltefosine’s entry into the *Leishmania* parasites relies on a P4-ATPase membrane transporter called miltefosine transporter (MT), which has as its main function the transport of phospholipids from the extracellular environment through the cell membrane.^18^ It is well-known that in vitro MF-selected parasites present a significant reduction in drug accumulation due to mutations in the *MT* gene, which leads to a defective MF transport machinery.^19,20^ However, this reduced drug uptake was also shown to be present in parasites naturally less susceptible to MF without significant differences in the *MT* gene sequence and/or expression of this transporter.^21,22^

Another protein plays a key role in MF transport, the MT’s beta subunit Ros3, which belongs to the Lem3p/CDC50 family.^23^ Together, they form the MT-Ros3 complex, and both of them are indispensable for the complex functionality.^24^ The Ros3 subunit has been shown to play a key role in phospholipid transport and susceptibility of yeast to MF and edelfosine.^25^ In *Leishmania* parasites, it has already been demonstrated that the absence or defects in Ros3 cause the retention of the whole MT-Ros3 complex in the endoplasmic reticulum and consequently resistance to MF.^24^ Moreover, polymorphisms in both *MT* and *ros3* genes were described as responsible for the reduced susceptibility to MF in an *L. infantum* clinical isolate.^15^

We previously reported that *L. braziliensis* clinical isolates from Brazilian patients exhibited differences in susceptibility to MF,^26^ as a result of differences in drug uptake.^21^ The levels of *ros3* mRNA were found to be decreased in tolerant isolates compared to a sensitive one, suggesting that a low abundance of this component of the MF transport complex could be the cause of a reduced susceptibility observed in tolerant isolates.^21^

Since gene and/or chromosome copy number variations have been implicated in the mechanisms of drug resistance and variation of susceptibility to drugs in *Leishmania*,^27,28^ we hypothesized that the differences in MF susceptibility, drug uptake, and *ros3* transcript abundance in these isolates might be the result of a differential *ros3* gene dosage in these parasites. Thus, in this work we investigated the copy number of the *ros3* gene in these *L. braziliensis* clinical isolates and the consequences of a differential *ros3* gene dosage for MF susceptibility by overexpressing and knocking out this gene in *Leishmania*.

## Results

### Differential *ros3* DNA Abundance among *L. braziliensis* Clinical Isolates

It was previously reported by our group that *L. braziliensis* clinical isolates obtained in Brazil presented variable susceptibility to MF.^26^ Analysis of these isolates through transcriptome sequencing and quantitative real-time reverse transcription polymerase chain reaction (RT-PCR) allowed the demonstration that the *ros3* mRNA abundance was upregulated in the most susceptible isolate (S) when compared to the M2903 reference strain (RS) and the two more tolerant isolates (T1 and T2)^21^ (Table 1).

**Table 1:** Susceptibility to MF and *ros3* Transcript Abundance in *L. braziliensis* Clinical Isolates

		EC_50_ ± SEM^a^ (μM)			
abbreviation^b^	identification code^c^	promastigotes	amastigotes	FC RNaseq^d^ (*p*-value)	FC real-time^e^ (*p*-value)
RS	MHOM/BR/1975/M2903	53.5 ± 6.6	2.7 ± 0.2	ND	–1.85 (0.0993)
S	MHOM/BR/2005/LTCP16012	22.9 ± 3.7	0.8 ± 0.1	1.0	1.0
T1	MHOM/BR/2006/LTCP 16907	101.2 ± 6.0	3.3 ± 0.4	–2.04 (0.0069)	–3.97 (0.0198)
T2	MHOM/BR/2009/LTCP 19446	90.4 ± 5.2	4.2 ± 0.2	–2.10 (0.0146)	–3.93 (0.0114)

a EC_50_ ± SEM of MF for promastigotes and amastigotes of *L. braziliensis* clinical isolates and M2903 reference strain. Data previously published in Espada et al.^26^

b Code used for strain and isolates used in this study.

c International code of each isolate.

d Fold-change (FC) in *ros3* transcript abundance in tolerant isolates relative to the abundance in the sensitive isolate and adjusted *p*-value evaluated by three biological replicates of each isolate and M2903 reference strain. (ND) Not differentially expressed in this transcriptome. Data previously published in Espada et al.^21^

e Normalized expression of *ros3* and adjusted *p*-value relative to S isolate assessed by quantitative real-time RT-PCR. Data previously described in Espada et al.^21^

Since the regulation of gene expression in trypanosomatids does not generally occur at a transcriptional level, a differential expression is mostly a result of differences in gene copy number or post-transcriptional regulation.^28,29^ Copy number variation (CNV) was assessed in these isolates by a quantification of the *ros3* DNA abundance by real-time PCR. Two housekeeping genes, *gapdh* and *tbp*, were chosen for normalization in these experiments after they showed a consistent expression between these isolates in the RNaseq data (data not shown).^21^

**Figure 1:**
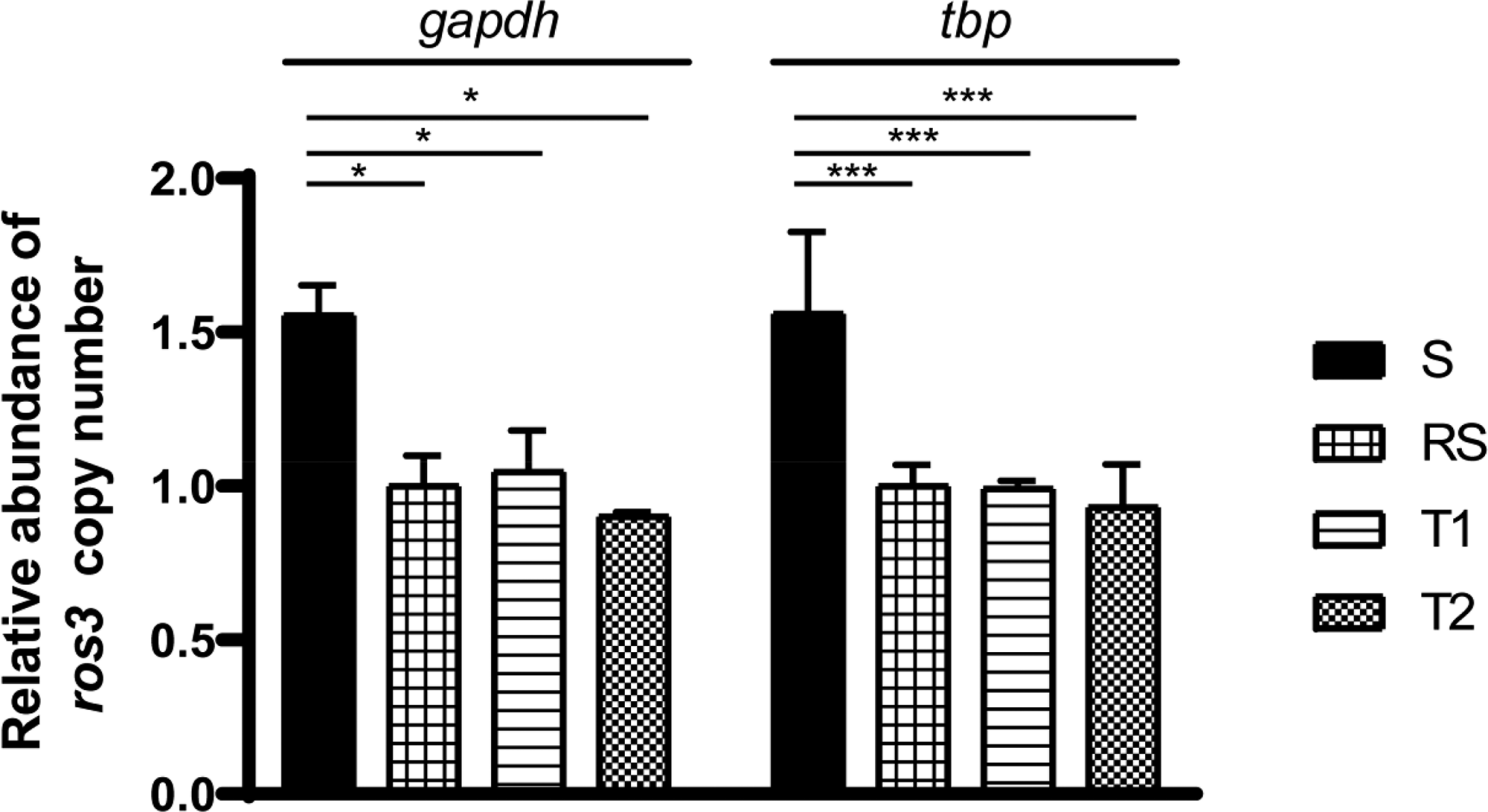
Relative abundance of *ros3* DNA in *L. braziliensis* clinical isolates and reference strain (RS). Each bar represents the mean *ros3* DNA molecules number in each isolate relative to the molecule numbers in RS. Relative abundance was assessed by real-time PCR in two independent experiments using two different normalizer genes (*gapdh* and *tbp*). Three independent biological replicates and three technical replicates were employed in each experiment. Statistical significance was determined using One-way ANOVA and Tukey’s multiple comparison test. (*) *p* < 0.05 and (**) *p* < 0.001.

Significant differences in the *ros3* DNA abundance were observed between S and T1/T2 isolates using both *gapdh* and *tbp* as normalizers (Figure 1). The most susceptible isolate (S) presented a significant 0.5-fold increased abundance of *ros3* DNA compared to the tolerant isolates in experiments employing different normalizer genes. The abundance profile of *ros3* DNA molecules correlated with the abundance of transcripts of this gene previously reported by Espada et al.^21^ and is highlighted in Table 1. Interestingly, the DNA quantification did not reveal differences between the reference and T isolates, in spite of the previously noted changes in half-maximal effective concentration (EC_50_) and mRNA abundance.

### Overexpressing *ros3* in the Tolerant Isolate Does Not Increase MF Susceptibility

The increased abundance of *ros3* DNA in the isolate S led us to investigate whether the addition of more copies of this gene in tolerant parasites would lead to an increase in susceptibility to MF. To test this hypothesis, we overexpressed the *ros3* gene in the isolate T2, which presented the highest EC_50_ to MF. The *ros3* coding sequence was amplified from the T2 genome, cloned downstream to an *L. tarentolae adenine phosphoribosyl* (*aptr*) and upstream to a *L. tarentolae calmodulin* (*camCB*) untranslated region (UTR). The generated SR construct was then linearized and delivered by electroporation for integration in the SSU *locus* of this isolate. Recipient parasites were the T2 isolate and *L. major* FV-1 (Lm), included to verify if these findings would be similar for another *Leishmania* species. After a selection with hygromycin B in solid M199 seven clones of T2 and eight clones of Lm were screened by PCR for the presence and correct integration of the SR insert (Figure S1).

**Figure 2:**
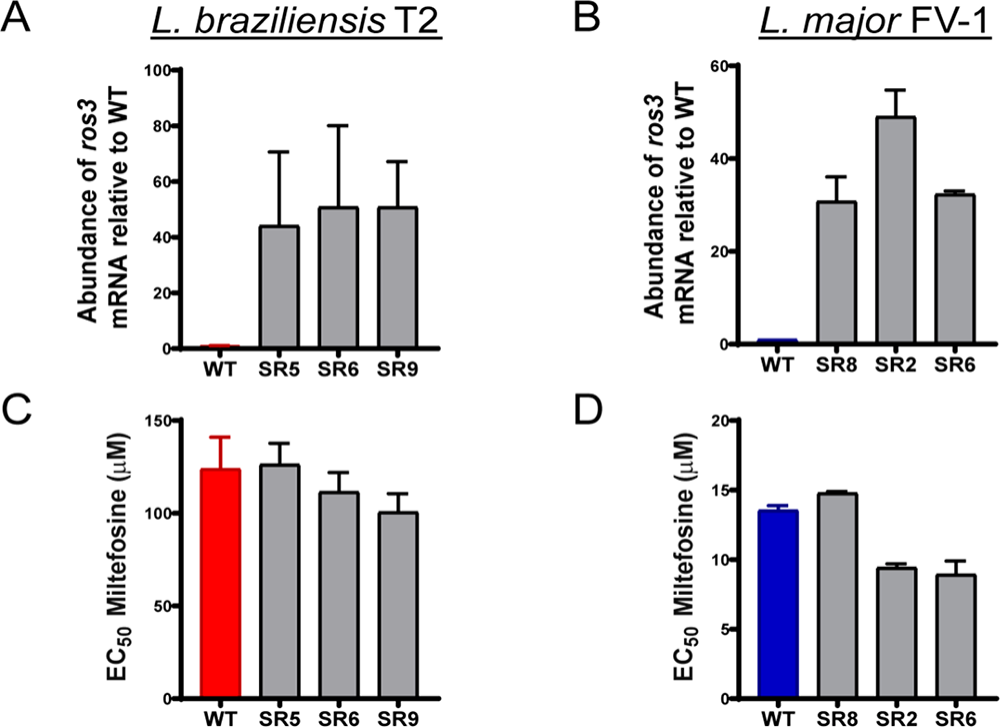
Relative abundance of the *ros3* gene mRNA and phenotypic characterization in SR clones. Abundance of the *ros3* mRNA was quantified in each SR clone and WT parasites of *L. braziliensis* T2 (A) and *L. major* FV-1 (B) by real-time qPCR. Normalization was done using *tbp* gene Ct values, and each bar represents the mean ± SEM abundance of the *ros3* transcript relative to WT parasites obtained in two independent experiments using two biological and three technical replicates (A, B). Susceptibility to MF was evaluated in SR clones and WT parasites of *L. braziliensis* T2 (C) and *L. major* FV-1 (D) by an MTT assay. Each bar represents the mean ± SEM EC_50_ to MF obtained in three independent experiments performed in triplicate.

Three random T2 and three Lm SR clones presenting the SR cassette integrated in the right orientation were selected for an *ros3* mRNA abundance quantification. The relative abundance of the *ros3* mRNA in each clone relative to the WT parasites was assessed through real-time qPCR using *tbp* as a normalizing gene for T2 and Lm lines (Figure 2). An increase in transcript abundance was observed in both species. In *L. braziliensis* T2 SR clones, levels of *ros3* mRNA were increased 44- to 50-fold when compared to WT parasites (Figure 2A). In *L. major* SR clones, the increase in *ros3* mRNA levels varied from 30.8- to 49.0-fold compared to the WT parasite (Figure 2B).

The effects of *ros3* overexpression were investigated by an evaluation of log-phase promastigotes MF susceptibility in SR clones of *L. braziliensis* by 3-[4,5-dimethyl-2-thiazolyl]-2,5-diphenyl-2*H*-tetrazolium bromide) (MTT). Compared to the EC_50_ determined for Lb WT (123.9 ± 17.08 μM), a nonstatistically significant decrease of 18% was observed for the clone SR9 (EC_50_ = 100.6 ± 9.94 μM) (Figure 2C).

In Lm SR clones the EC_50_ reduction was more pronounced and significant for two out of three clones. A 30% decrease in EC_50_ values was observed for clone SR8 (8.93 ± 0.97 μM) as compared to the EC_50_ calculated for the WT parasite (13.54 ± 0.34 μM) (Figure 2D). However, no significant correlation between the *ros3* mRNA abundance and MF susceptibility was found for either *L. braziliensis* or *L. major ros*3 overexpressor clones (*r* = −0.800 and *p* = 0.333; *r* = −0.600 and *p* = 0.4167, Spearman’s correlation test, respectively).

Increasing the hygromycin selection pressure from 32 to 128 μg/mL did not lead to a more pronounced reduction in EC_50_ values in either species. In T2 SR clones, the EC_50_ of SR2 and SR5 decreased from 111.53 and 126.64 μM to 102.3 and 119.5 μM, respectively. In Lm SR clones the EC_50_ of SR2 and SR8 under increased hygromycin B pressure changed from 10.8 and 14.7 to 13.5 and 16.5 μM, respectively.

### Partial or Complete Removal of ros3 Reduces Susceptibility and Uptake of MF in L. major

As an alternative strategy to evaluate the role of the *ros3* gene dosage effect on MF susceptibility, an Lm strain constitutively expressing Cas9 and T7 RNA polymerase (Lm Cas9/T7) was used to generate mono (partial) and diallelic (complete) knockouts for *ros3* using CRISPR/Cas9.

For that, small guide RNAs (sgRNAs) coding template and donor DNAs were generated by PCR in vitro and delivered to the parasite, driving the Cas9-mediated break and incorporation of the donor DNA containing a blasticidin resistance (BlastR) gene by homologous recombination, replacing the complete *ros3* open read frame (ORF).

**Figure 3:**
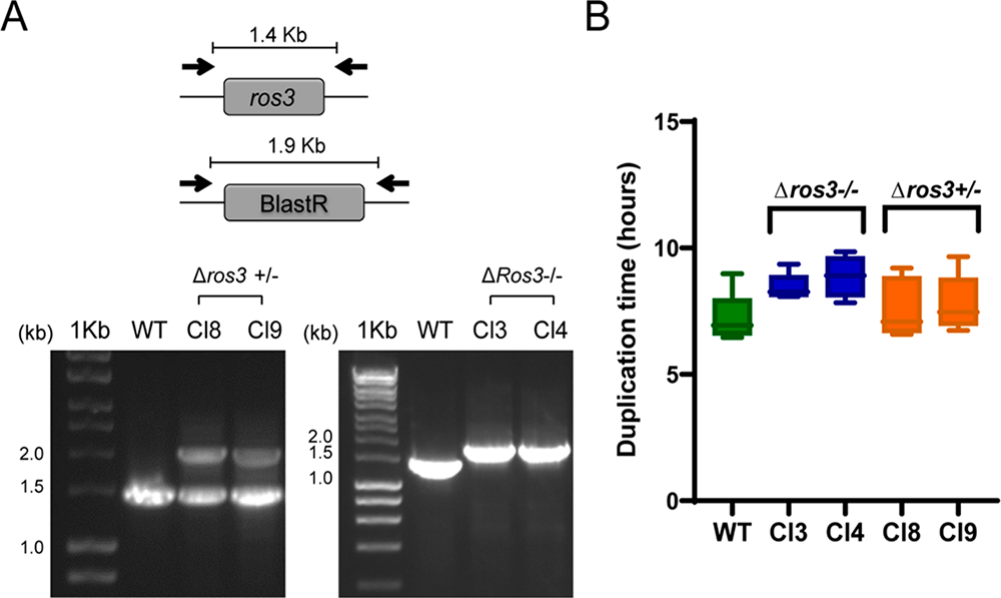
*ros3* knockout verification in recovered clones. (A) Schematic representation of the strategy used for ORF KO verification in blasticidin-resistant clones. In diallelic knockouts, *Blast*-R was amplified and resulted in a 1.9 kb fragment, whereas in monoallelic *ros*3 KOs both *Blast*-R and *ros3* are amplified resulting in two bands of 1.9 and 1.4 kb, respectively. (B) Parasite doubling time for each incomplete (Δ*ros3* +/–±) and complete (Δ*ros3* −/−) KO clones and for WT *L. major* Cas9/T7. Each box represents the mean ± SEM of the duplication time evaluated during 4 d.

After the selection with blasticidin, single and complete knockouts (KOs) for the *ros3* gene were confirmed by PCR in the heterogeneous parasite population that was previously cloned by serial dilution (Figure 3). To verify the presence or absence of *ros3* in the clones recovered from *ros3* KO experiments, primers annealing outside the ORF were used. The presence of the *ros3* gene was identified by the presence of a 1.4 kb fragment, whereas its substitution by a *Blast*-R gene resulted in the amplification of a 1.9 kb fragment (Figure 3A). Six clones with incomplete and six clones with complete deletions of *ros3* (Figure 3) were identified. Two incomplete (Cl8 and Cl9) and two complete (Cl3 and Cl4) *ros3*-deficient mutants were selected for MF susceptibility and uptake characterizations.

**Figure 4:**
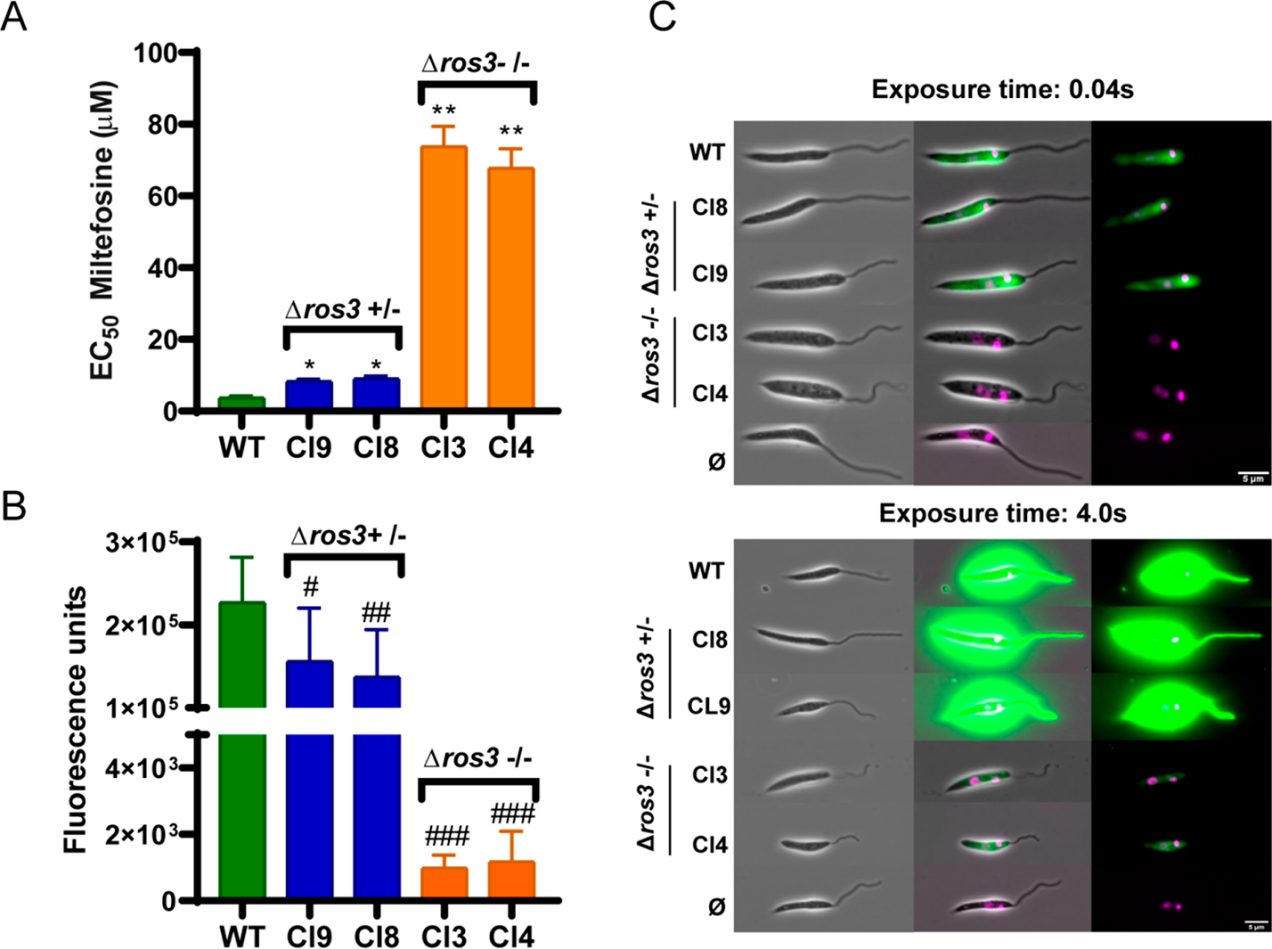
Susceptibility and uptake of MF in *L. major ros3* mono- and diallelic KOs. (A) The EC_50_ of MF was determined for the *L. major* Cas9/T7 strain and for mono- and diallelic knockouts by MTT. Mean and SEM results of three or more independent experiments were calculated. (*) *p* < 0.005. (**) *p* 0.0001. (B) Uptake of MT-EtBDP in monoallelic (Cl8 and Cl9) and diallelic (Cl3 and Cl4) *ros3* knockouts. (#) *p* < 0.01; (##) *p* < 0.001; (###) *p* < 0.0001. Uptake of MT-EtBDPY was evaluated by flow cytometry. A reduction in fluorescence (FLH-1) inside parasites was observed in *ros3* incomplete knockouts. In complete knockouts, the reduction was even higher. (C) Evaluation of MT-EtBDP uptake in WT (Lm Cas9/T7) and in *ros3* monoallelic (Cl8 and Cl9) and diallelic (Cl3 and Cl4) knockouts by fluorescence microscopy. Different exposure times of 0.04 and 4 s were used to confirm the presence of the labeled MF inside parasites. As a background, the control WT Cas9/T7 incubated without MT-EtBDPY was exposed to the same conditions for image acquisition.

To exclude the possible interference of growth rates between the mutants in the viability assays, the doubling time of the generated lines in parallel with the WT parasites was determined as described in the Methods Section. Although a mild increase in the doubling time was observed in complete knockouts (Cl3 and Cl4), this difference was not significant [analysis of variance (ANOVA) and Tukey’s multiple comparisons test] (Figure 3). Other features including size, shape, or motility were visually inspected under a bright field-inverted microscope. None of the clonal mutant population cultures presented differences detectable to the human eye (Figure 4C).

The susceptibility of partial and complete knockouts to MF was determined by MTT. Initially, the susceptibility of *L. major* Cas9/T7 was compared to the *L. major* WT line (the background in which *L. major* Cas9/T7 was generated) in order to evaluate if the modified parasite could behave differently regarding MF susceptibility. No significant differences were observed between Lm WT (EC_50_ = 6.57 ± 0.89 μM) and the Lm WT Cas9/T7 strain (EC_50_ = 3.46 ± 0.48 μM) (non-parametric t-test).

When compared to Lm Cas9/T7 WT parasites, a significant approximately threefold reduction (*p* > 0.002) in EC_50_ values for MF was observed for monoallelic deleted *ros3* mutants (Table 2). When the two alleles of *ros3* were removed (diallelic deleted *ros3* mutants), a more pronounced reduction in the susceptibility to MF (∼20-fold reduction) was detected (*p* < 0.0001) (Table 2 and Figure 4A).

**Table 2:** Susceptibility of *L. major ros3* Nono- and Diallelic Knockouts to MF

sample	EC_50_ ± SEM^a^ (μM)	activity index^b^ (AI)
Lm WT	6.57 ± 0.89	
Lm WT Cas9/T7	3.46 ± 0.48	
Δ*ros3* ± Cl8	10.22 ± 1.27	2.95
Δ*ros3* ± Cl9	9.66 ± 1.43	2.79
Δ*ros3* −/– Cl3	73.82 ± 5.53	21.33
Δ*ros3* −/– Cl4	71.47 ± 5.49	20.65

a EC_50_ ± SEM of MF for promastigotes determined by MTT assay.

b Activity index (AI) was calculated by dividing the EC_50_ of each clone by the EC_50_ of the reference parasite *L. major* Friedlin Cas9/T7.

The uptake of MF in mono- and diallelic knockouts was determined by flow cytometry using MF labeled with 11-(4′,4′-difluoro-6′-ethy[11-(4′,4′-difluoro-6′-ethyl-1′,3′,5′,7′-tetramethyl-4′-bora-3′a,4′a-diaza-s-indacen-2′-yl)-undecylphosphocholine] (MT-EtBDPY) and compared to Lm Cas9/T7 WT. In the absence of one allele of *ros3*, the amount of fluorescence inside the parasite presented a mild but significant reduction. However, in *ros3* diallelic knockouts, the uptake is reduced 100-fold compared to that of WT Cas9/T7. These data suggested that, in the absence of one or two copies of *ros3*, the MF entry in *L major* is impaired (Figure 4B).

The drug uptake variation was also demonstrated by fluorescence microscopy (Figure 4C). With 0.04 s of exposure, no fluorescence was observed inside Ros3 null mutants, while positive labeling was seen in WT parasites and incomplete knockouts. However, after longer exposures (4 s), a weak MF fluorescence signal was observed in complete knockouts, demonstrating that some MF uptake happened even in the complete absence of *ros3* (Figure 4C).

## Discussion

The susceptibility to MF was found to be variable among *L. braziliensis* clinical isolates, raising the concern of intrinsic tolerance in isolates circulating in Brazil.^26^ A further investigation of the mechanisms behind the differential susceptibility to MF in these isolates revealed differences in drug uptake and in the abundance of the *ros3* transcript, an essential component of the MF transport machinery.^21,24^ Considering the nature of gene expression regulation in *Leishmania*,^30^ the high genome plasticity,^31,32^ and the previous association of gene copy number variation (CNV) with drug resistance in these organisms,^33^ in this work we investigated whether or not a differential *ros3* mRNA abundance in *L. braziliensis* clinical isolates was related to variability in *ros3* gene dosage. Furthermore, using different DNA manipulation approaches we evaluated if susceptibility to MF could be modulated by the addition or removal of *ros3* gene copies, mimicking a CNV condition.

A 0.5-fold increase in the *ros3* DNA abundance was found in the *L. braziliensis* susceptible isolate when compared to tolerant isolates and the reference strain. This indicated the presence of an extra copy of *ros3*, the most likely reason for the increased abundance of *ros3* mRNA observed. The *ros3* extra copy may represent an isolated event of gene duplication or a chromosome 32 tetrasomy, but further investigation about the chromosome content in these isolates has not been performed yet.

These findings then led us to investigate whether an alteration in the *ros3* gene dosage was enough to modulate the susceptibility to MF. On the one hand, the integration of an extra *ros3* copy in the genome significantly increased the mRNA abundance in overexpressing clones. However, the accumulation of *ros3* transcripts did not lead to significant changes in the MF susceptibility in these parasites, suggesting that the increase of *ros3* transcripts alone was not capable of modulating the MF susceptibility in *L. braziliensis* T2 and *L. major*. On the other hand, the generation of mono- and diallelic *ros3* knockouts using CRISPR/Cas9 in *L.major* led to 2-fold and 20-fold increases in the EC_50_ to MF, respectively.

The observation of the unchanged susceptibility to MF in *L. braziliensis* T2 and in *L. major* was surprising. The expression system employed for *ros3* overexpression herein (pLEXSY) is capable of inducing the expression of exogenous and endogenous genes in different *Leishmania* species, including *L. braziliensis*.^34−37^ Moreover, differential *ros3* and *MT* expressions have already been shown by others to play a role in the susceptibility of *Leishmania* to MF.^22^ An *L. braziliensis* Peruvian isolate and the Brazilian reference strain M2904 were shown to be 6–10-fold less susceptible to MF when compared to *L. donovani* due to a reduced expression of Ros3 in the plasma membrane of *L. braziliensis*.^22^ This is the same range of variation in susceptibility to MF encountered among Brazilian *L. braziliensis* clinical isolates.^26^ Additionally, the same study showed that *L. braziliensis* overexpressing *ros3* demonstrated a 3.5-fold reduction in MF EC_50_. Importantly, they have shown that, in this context of *ros3* overexpression in *Leishmania*, there is an increase not only in Ros3 protein abundance in plasma membrane but also of MT protein, suggesting that *ros3* overexpression triggers an endogenous *MT* overexpression, since the complete MT-Ros3 complex is essential for MF uptake.^22^ It is possible therefore that the susceptibility of Ros3 overexpressor mutants was unchanged because they lacked the necessary MT to compound the transporter complex MT-Ros3.

However, one important limitation in this study is the lack of a demonstration of an increased abundance of the Ros3 protein in the overexpressing mutants. Various attempts of immunodetection and protein tagging did not produce clear-cut results, so this remains to be achieved. Therefore, we must consider biological factors that could explain the lack of phenotype in the overexpressor mutants. An overexpression was achieved using the *L. braziliensis* T2 *ros3* coding sequence upstream to a heterologous UTR element. The lack of *ros3* UTR elements may have hampered a proper mRNA processing and translation.^38^ Where overexpression in *L. major* is concerned, the limited identity (72%) between *L. braziliensis* and *L. major ros3* coding sequences could potentially lead to interference in Ros3 folding, interaction with MT, and membrane insertion when expressed in *L. major*.^23^

Moreover, besides *ros3*, another 35 genes were shown to be differentially expressed between sensitive and tolerant isolates,^21^ and those could represent indispensable partners for an effective change in the MF uptake. Therefore, increasing the *ros3* transcript abundance through the methodology employed herein was not enough to sensitize *L. braziliensis* T2 and *L. major* to MF.

However, results obtained employing a loss of function approach revealed that the knockout of *ros3* modulates the susceptibility and uptake of MF in a gene-dosage-dependent way. The generation of complete and incomplete *ros3* knockout in *L. major* caused a significant decrease in uptake and in the susceptibility to MF, suggesting that the presence of this gene is critical for the susceptibility to MF. Similar results were observed in *L. donovani*, which presented a reduction in the susceptibility to MF of 1.7- and 14.2-fold in *ros3* mono- and diallelic knockouts, respectively, suggesting that the MF tolerance phenotype caused by the reduction in *ros*3 gene dosage is not a species-specific phenotype. Interestingly, these values are also comparable to the reduction of 1.9- and 13.7-fold observed in the context of MT mono- and diallelic knockouts, which reinforces the codependence of both proteins.^24^

In addition, the complete removal of *ros3* did not abolish MF internalization completely, as residual fluorescent MF was observed inside the diallelic knockouts, suggesting that other routes for the internalization of MF, such as endocytosis or diffusion after incorporation into cell membranes, may be involved, even if poorly.^39^ If in the absence of the MT-Ros3 complex, diffusion through the membrane occurs in significant levels, variations in the composition and structure of plasma membrane in *Leishmania* parasites might also play a role in a differential susceptibility to MF.

To the best of our knowledge, this is the first demonstration of *ros3* gene dosage described for *Leishmania* clinical isolates associated with a differential susceptibility and uptake of miltefosine. However, the dependence of the MT-Ros3 complex for MF transport has been repeatedly shown as the Achille’s heel of MF efficacy in *Leishmania* parasites either by acquisition of inactivating mutations in these genes^15,19,20,40,41^ or by a differential expression of this complex in *Leishmania* plasma membrane.^22^

Taken together, these results reinforced the role of the Ros3 subunit as a limiting factor for the MF uptake in *Leishmania* parasites and demonstrated for the first time that the *ros3* gene dosage plays a role in a differential susceptibility to MF not only in *L. braziliensis* isolates never exposed to MF but also in *L. major*.

The high cure rates,^42,43^ together with the high intracellular concentrations of MF achieved during therapy^44,45^ and the low number of cases of resistant parasites recovered after treatment with MF,^13,15^ are good indicatives that the variations observed in these *L. braziliensis* isolates are not enough to cause a treatment failure. However, it is important once again to highlight that MT-Ros3 is repeatedly being described as the cause of susceptibility reduction, not only in parasites selected in vitro under drug pressure but also in an isolate recovered after VL treatment with MF failure.^15^ In this scenario, our findings highly encourage the search for new drug therapy schemes, such as drug combinations that could enhance the MF activity, or even modifications in MF molecule that could promote the entrance of the drug by an alternative route in an attempt to avoid selection of resistant parasites and loss of the only effective oral drug for leishmaniasis treatment.

## Conclusions

Being the only oral drug currently in use for leishmaniasis treatment, preventing a loss of MF due to resistance is a necessary effort. Our study reinforce the role of MT-Ros3 machinery in MF resistance by showing the direct effect of *ros3* gene dosage in MF susceptibility and uptake not only in long-term laboratory-cultured *Leishmania* reference strains or parasites selected for resistance but also in *L. braziliensis* clinical isolates not previously exposed to MF. Our results encourage the search for new variants of MF molecule, different drug-delivery systems, or even coadministration with other molecules that could enhance MF transport and overcome the stringent dependence of active transport through the MT-Ros3 complex.

## Methods

### Chemical Compounds

Miltefosine and MTT were purchased from Sigma-Aldrich and diluted in sterile water and phosphate-buffered solution (PBS), respectively. The BODIPY-labeled MF MT-EtBDP was kindly donated by Dr. A. U. Acuña (Instituto de Química-Física “Rocasolano”, CSIC) and prepared as described.^46^ Hygromycin B and blasticidin S hydrochloride were purchased from Melford Laboratories Ltd.

### Cultivation of Leishmania Parasites

The cell lines used in this work were: the parental strain of *L. major* Friedlin FV-1 (MHOM/IL/1980/Friedlin) (Lm) and the modified Cas9/T7-expressing *L. major* Friedlin FV-1 (Lm Cas9/T7);^47^ the *L. braziliensis* reference strain (RS) (MHOM/BR/1975/M2903) and three *L. braziliensis* Brazilian clinical isolates, namely, MHOM/BR/2005/LTCP16012 (named S, for Sensitive), MHOM/BR/2006/LTCP16907 (T1, for Tolerant 1), and MHOM/BR/2009/LTCP19446 (T2, for Tolerant 2). The susceptibility of these isolates to MF was previously reported^26^ (Table 1).

Promastigotes of *Leishmania* were cultivated at 28 °C in M199 medium (Sigma-Aldrich) supplemented with 2.2 g/L NaHCO_3_, 0.005% hemein, 40 mM 4-(2-hydroxyethyl)piperazine-1-ethanesulfonic acid (HEPES), pH 7.4, and 10% heat-inactivated fetal calf serum (FCS). For *L. braziliensis*, 2% male urine was added to the culture. Transfectants were maintained in MM199 media, which was made by diluting M199 media powder and supplementing with 2.2 g/L NaHCO_3_, 0.0025% hemein, 40 mM HEPES, pH 7.4, 0.1 mM adenine hemisulfate, 1.2 μg/mL biopterin, and 20% FCS. The appropriate selection drug was added at 32 μg mL^–1^ hygromycin B or 5 μg mL^–1^ blasticidin S hydrochloride.

### Generation of Parasites Overexpressing of *ros3* Gene

For the *ros3* overexpression in the *L. braziliensis* T2 isolate and *L. major* Friedlin FV-1 strain, the open reading frame of the gene *ros3 (LbrM.32.0580*) was amplified from *L. braziliensis* T2 total DNA using the primer pair SR_*Bgl*II-Fow and SR_*Not*I-Rev (Table S1) and cloned into the plasmid pLEXSY-hyg2 (Jena Biosciences). In this expression vector, the gene of interest is under the regulation of *L. tarentolae adenine phosphoribosyl transferase* (*aprt*) UTR at 5′ and *calmodulin* (*camCB*) UTR at 3′.^48^ The generated construct (SR) was then linearized with the restriction enzyme *Swa*I, and purified cassette pLEXSY-hyg2-SR was delivered to *L. braziliensis* T2 isolate and *L. major* Friedlin FV-1 parasites for integration in the small subunit of rDNA (SSU) by electroporation, as previously described.^49^ Transgenic parasites overexpressing the *ros3* gene (SR clones) were selected in a semisolid M199 medium supplemented with 32 μg/mL hygromycin B as described.^50^ Recovered clones were then maintained in liquid M199 in the presence of 32 μg/mL hygromycin B. Genomic DNA was extracted using the protocol described by Rotureau et al.,^51^ and integration of the SR cassette into the SSU rDNA *locus* was confirmed by PCR using the primers provided with pLEXSY-hyg2 F3001, A264, F3002, and A384 (Jena Bioscience) (Table S1).

### Quantitative Real-Time PCR

The abundance of *ros3* mRNA and DNA in SR clones and isolates was quantified by real-time RT-PCR (qPCR) using total RNA or genomic DNA as templates, respectively. For the mRNA quantification, cDNA was synthesized from total RNA using MuLV Reverse Transcriptase (Applied Biosystems). Briefly, 6 μg of DNase-treated RNA was incubated with 1 μg of random primers (Thermo Fischer Scientific) for 10 min at 70 °C. After this period 1X MulV-RT buffer, 0.01 mM dithiothreitol (DTT), 5.5 mM MgCl_2_, and 1 mM dNTPs were added to the system and incubated at 42 °C for 2 min. Reverse Transcriptase was then added to the RT+ tubes but not the RT– tubes (control of DNA absence), and the reaction was incubated at 25 °C for 10 min, followed by incubations at 48 °C for 30 min and at 95 °C for 5 min according to the manufacturer’s instructions.

One hundred nanograms of in vitro synthesized cDNA was used a as template for qPCR, which was performed in a StepOne Plus System (Applied Bios Systems) using SYBR Green PCR Master Mix (Thermo Fisher Scientific). The following program was used: 95 °C for 10 min followed by 40 cycles at 95 °C for 15s, 60 °C for 60 s, and 72 °C for 20 s. The 163 base pair (bp) *ros3* gene fragment was amplified using the primer pair LbLm_Ros3-F and LbLm_Ros3-R (Table S1). The housekeeping g*lyceraldehyde 3-phosphate dehydrogenase* (*gapdh*) and *tata-box-binding protein* (*tbp*) coding genes were used for normalization and amplified using the primer pair *gapdh*-F and *gapdh*-R and Lb_*tbp*-F/Lm_*tbp*-F and LbLm_*tbp-*R, respectively (Table S1). Three biological replicates and three technical replicates of each sample were evaluated for *ros3* and *gapdh*/*tbp* mRNA and DNA abundance determination. The threshold cycle (Ct) obtained for *ros3* in each sample was normalized by the Ct of the *gapdh*/*tbp* genes. The 2^–ΔΔCt^ equation was used to determine the expression of *ros3* genes relative to RS, in the case of isolates, or to the wild type (WT) parasites when SR clones were characterized, respectively.^52^ 2^–ΔΔCt^ values were then plotted on GraphPad Prism 6, and statistical analyses were performed using a one-way ANOVA analysis followed by Tukey’s multiple comparison tests.

For quantifying the copy number of the *ros3* gene, the samples were submitted to real-time PCR together with a standard curve using the pGEM-T-Ros3-M2903 plasmid (previously available in the laboratory).^21^ A linear regression of Ct values and *ros3* molecule number was constructed based on the data obtained for the standard curve and employed to determine the number of molecules in each sample. After normalization by *tbp* or *gapdh* molecule values, the relative abundance of *ros3* was calculated by dividing the normalized amount of *ros3* in each isolate by the amount in RS.

### Generation of Mono- and Diallelic Knockouts for the *ros3* Gene

Knockout mutants of this study were generated by CRISPR/Cas9 technology and the LeishGEdit toolkit on the background of *L. major* overexpressing Cas9/T7.^53^ Primer sequences for the PCR generation of sgRNA templates and donor DNAs for the *ros3* gene ID (*LmjF.32.0510*) were selected using LeishGEdit (http://www.leishgedit.net/).^53^

The sgRNA templates for target gene cleavage were generated by PCR reactions using the G00 primer together with the 5′ (LmRos3_5′sgRNA) or 3′sgRNA (LmRos3_3′sgRNA) LeishGEdit primers in individual tubes (Table S1). Donor DNA for generation of *ros3* knockouts was also obtained by PCR reactions using the pTBlast_v1 plasmid as a DNA template and Upstream Forward Primer (LmRos3_UFP) and Downstream Reverse Primer (LmRos3_DRP) initiators (Table S1). Detailed protocols used for PCR reactions are described in Beneke and Gluenz.^53^

The delivery of sgRNA templates and donor DNA to 1 × 10^7^ Lm Cas9/T7 log-phase promastigotes was done by a transfection with Amaxa Nucleofector using program X-001 in a transfection buffer as previously described.^53^ After transfections, parasites were added to flasks containing MM199, and after 6 h, 5 μg/mL blasticidin was added to the culture.^53^ After two splits in 1:100 proportion, the recovered population was cloned in 96-well plates in three different proportions 0.1, 1.0, and 10 promastigotes/ml. Population was considered to be clonal when no more than 30% of the wells in each dilution presented growth.

Monoallelic (single) and diallelic (double) knockouts were verified through PCR reactions using primers LmRos3_UTR-F and LmRos3_UTR-R, which anneal outside the *ros3* ORF. The presence of WT *ros3* results in the amplification of a 1.4 kb fragment, whereas the substitution by the *Blast*-R gene would result in the amplification of a 1.9 kb fragment. DNA obtained from the *ros3* transfectant population and of Lm Cas9/T7 were used as positive controls for partial KO and target gene presence, respectively.

### Doubling Time Measurement

For doubling time characterization in mutants and WT parasites, the culture density was adjusted to 1 × 10^6^ promastigotes/mL in M199. After an incubation for 24 h at 28 °C, the cell culture density was determined using a cell counter (CASY model TT, Roche Diagnostics) with a 60 μm capillary and exclusion of particles with a pseudo diameter below 2.0 μm. The cell density was adjusted again to 1 × 10^6^ promastigotes/mL in a new flask. This procedure was repeated for 4 d. The doubling time (DT) was calculated using the following formula.
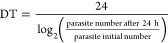
1

### Susceptibility Assays

The susceptibility to MF was determined by an MTT assay.^54^ Briefly, 2 × 10^6^ (*L. braziliensis)* or 2 × 10^5^ (*L. major*) log-phase promastigotes were incubated in the presence of increasing concentrations of MF for 24 h (SR clones) or 48 h (knockout mutants). MF concentrations employed for *L. braziliensis* were 400, 280, 240, 200, 140, 120, 100, 70, 60, 50, and 35 μM, and for *L. major* they were 120, 90, 80, 70, 60, 45, 30, 15, 7.5, 3.75, and 1.875 μM. The cell viability was then determined by an incubation with 5 mg/mL MTT followed by cell lysis with 4% SDS and optical density (OD) measurement at 690 and 595 nm. OD values were then converted in EC_50_ values by sigmoidal regression curves using GraphPad Prism 6 software. Susceptibility assays were conducted in triplicate, and at least three independent experiments were performed.

### Uptake of MT-EtBDPY

The uptake of MT-EtBDPY was evaluated as described in Espada et al.^21^ Briefly, log-phase *Leishmania* promastigotes were incubated in HEPES-NaCl buffer (21 mM HEPES, 137 mM NaCl, 5 mM KCl, 0.7 mM NaH_2_PO_4_, 6 mM glucose, pH 7.05) supplemented with 0.3% (w/v) bovine serum albumin (BSA) and 500 μM phenylmethylsulfonyl fluoride (PMSF) (Sigma-Aldrich) for 15 min at 28 °C. After this period, 1 μM MT-EtBDP was added, and the incubation was continued for 5 min at 28 °C. Parasites were washed three times with HEPES-NaCl containing 0.3% BSA to remove the noninternalized labeled molecules. The parasites were then suspended in PBS, and the fluorescence intensity was measured using a BD Accuri C6 flow cytometer (BD Biosciences). Statistical analyses were performed using one-way ANOVA followed by Tukey’s multiple comparison tests using Graph Pad Prism 6. Values are reported with the standard error of measure (SEM).

### Fluorescence Microscopy

For MT-EtBDP uptake analysis, after noninternalized molecules were washed, a fraction of the parasites was incubated in PBS with 10 μg/mL Hoechst 33342. Parasites were pelleted, suspended in PBS, and then placed on a microscope slide inside a small area marked with a liquid blocker pen. A coverslip was applied, and the living cells were immediately imaged in a Zeiss Axioimager.Z2 microscope with a 63× numerical aperture (NA) 1.40 oil immersion objective and a Hamamatsu ORCA-Flash4.0 camera. The filters used for Hoechst 33342 and MT-EtBDP were 350/461 nm (excitation/emission) and 527/536 nm, respectively. As a background control, WT Cas9/T7 untagged and/or that did not receive the ligands or fluorescent molecules were imaged using the specific filters at the same exposure time (0.04 and 4 s). Images were processed using Fiji.^55^

## Supporting Information Available

- PCR confirmation of SR cassette for *ros3* overexpression in *L. braziliensis* T2 and *L. major* FV-1 clones (PDF)
- List of all oligonucleotides used in this work (PDF)
