# Supplementary material for: Ros3 (Lem3p/CDC50) Gene Dosage Is Implicated in Miltefosine Susceptibility in Leishmania (Viannia) braziliensis Clinical Isolates and in Leishmania (Leishmania) major
Source: ACS Infect Dis. 2021 Mar 16;7(4):849–58. doi: 10.1021/acsinfecdis.0c00857 (PMC8042657; doi:10.1021/acsinfecdis.0c00857)
Supplement: Supplementary file 1 — id0c00857_si_001.pdf [file id0c00857_si_001.pdf]

Supporting Information:

Ros3 (Lem3p/CDC50) gene dosage is implicated in miltefosine susceptibility in *Leishmania (Viannia) braziliensis* clinical isolates and in *Leishmania (Leishmania) major*

Caroline R. Espada[a,*], Andreia Albuquerque-Wendt[b,c,e], Valentín Hornillos[d], Eva Gluenz[b,e], Adriano C. Coelho[f], Silvia R. B. Uliana[a]

[a] Departamento de Parasitologia, Universidade de São Paulo, São Paulo, Brazil.

[b] Sir William Dunn School of Pathology, University of Oxford, Oxford, United Kingdom.

[c] Global Health and Tropical Medicine (GHTM), Instituto de Higiene e Medicina Tropical (IHTM), Universidade de Lisboa (UNL), Lisboa, Portugal.

[d] Departamento de Química Orgánica, Universidad de Sevilla and Centro de Innovación en Química Avanzada (ORFEO-CINQA), Sevilla, Spain.

[e] Wellcome Center for Integrative Parasitology, Institute of Infection, Immunity & Inflammation, College of Medical Veterinary and Life Sciences, University of Glasgow, Glasgow, United Kingdom.

[f] Departamento de Biologia Animal, Instituto de Biologia, Universidade Estadual de Campinas, Campinas, Brazil.

[*]Corresponding author: Caroline Ricce Espada. Departamento de Biologia Celular e Molecular e Bioagentes Patogênicos, Faculdade de Medicina de Ribeirão Preto, Universidade de São Paulo. Av. Bandeirantes, 3900, CEP 14049-900, Ribeirão Preto, SP – Brasil. Tel: +55-16-33153053; E-mail: caroline.respada@usp.br.

Number of pages: 3

Number of tables: 1

Number of figures: 1

Table S1 – List of oligonucleotide sequences used in this work

Name	Sequence
SR_*Bgl*II-Fow	CCCCAGATCTATGGTGGATCTAACCCCTAAGC
SR_*Not*I-Rev	CCCCGCGGCCGCCTTTGTATATCTTGGCATAACGAAGCAC
F3001	GATCTGGTTGATTCTGCCAGTAG
A264	CATCTATAGAGAAGTACACGTAAAAG
F3002	CTGCAGGTTCACCTACAGCTAC
A384	CCGATGGCTGTGTAGAAGTACTCG
LbLm_Ros3-F	ACGGCACACTCATCTGCGAC
LbLm_Ros3-R	CCAGCACTCCACATCGGACC
gapdh-F	TGCGACCAGGACCTTAT
gapdh-R	TGGGCTGCTCTTCATAGA
Lb_*tbp*-F	GCTTGGATCTGGACGAACTC
Lm_*tbp*-F	CCACCCATCAAGAACGTGCAAGAG
LbLm_*tbp*-R	GAGGTTAATGCCGACTGGAA
G00	AAAAGCACCGACTCGGTGCCACTTTTTCAAGTTGATAACGGACTAGCCTTATTTTAACTTGCTATTTCTAGCTCTAAAAC
LmRos3_5'sgRNA	GAAATTAATACGACTCACTATAGGTTTCTGCTGGTGTTACTCGAGTTTTAGAGCTAGAAATAGC
LmRos3_3'sgRNA	GAAATTAATACGACTCACTATAGGCCCACCCGATGCTCACTATTGTTTTAGAGCTAGAAATAGC
LmRos3_UFP	TTTCTTGCGCATTGGTTTTCATTCGTTGTTGTATAATGCAGACCTGCTGC
LmRos3_DRP	ATCATGAACGTCTGCAGCACAAGGTAACCGCCAATTTGAGAGACCTGTGC
LmRos3_UTR-F	GAAAGCGTCCCACACAAGAT
LmRos3_UTR-R	TCTCTGTCTCCAGGCTCCAT

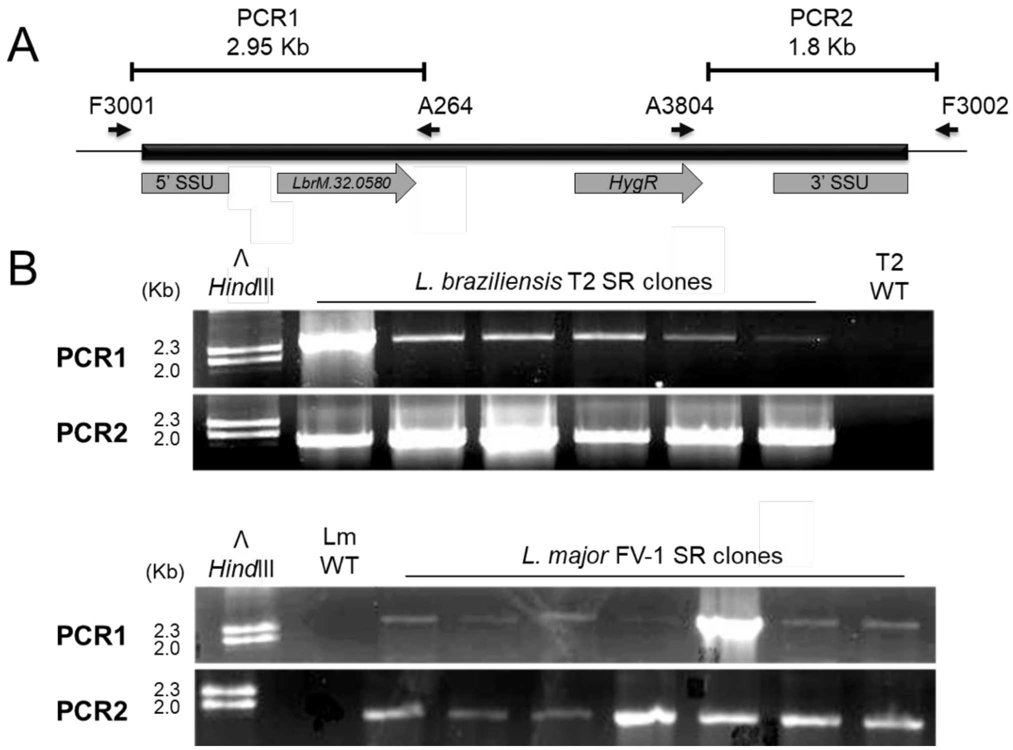

Figure S1 - Screening of *L. braziliensis* T2 and *L. major* FV-1 clones for the presence and correct integration of the SR cassette by PCR. (A) Schematic representation of expected integration region and localization of primers used for colony screening (black arrows). PCR1 primers annealed upstream to the 5' SSU integration site and downstream *Ros3* ORF (*LbrM.35.0580*) amplifying a 2.95 Kb fragment. PCR2 primers annealed in HygR (hygromycin resistance) gene and downstream to the 3' SSU integration region amplifying a 1.8 Kb fragment. (B) Both PCR1 and PCR2 were positive in all T2 and Lm transfectant clones, but not in corresponding wild-type (WT) parasites (non-transfected).
